# Bariatric surgery in individuals with type 2 diabetes is not associated with short or long-term risk of diabetic retinopathy progression: results from a nationwide cohort study

**DOI:** 10.1007/s00592-023-02140-w

**Published:** 2023-07-08

**Authors:** Anne S. Thykjær, Louise Rosengaard, Nis Andersen, Jens Andresen, Toke Bek, Javad Hajari, Steffen Heegaard, Kurt Højlund, Ryo Kawasaki, Caroline S. Laugesen, Sören Möller, Frederik N. Pedersen, Katja C. Schielke, Lonny Stokholm, Jakob Grauslund

**Affiliations:** 1 Department of Ophthalmology, Odense University Hospital, J. B. Winsløws Vej 4, 5000 Odense C, Denmark; 2 Department of Clinical Research, University of Southern Denmark, Odense C, Denmark; 3 Steno Diabetes Center Odense, Odense University Hospital, Odense C, Denmark; 4 Organization of Danish Practicing Ophthalmologists, Copenhagen, Denmark; 5 Department of Ophthalmology, Aarhus University Hospital, Aarhus, Denmark; 6 Department of Ophthalmology, Rigshospitalet-Glostrup, Copenhagen, Denmark; 7 Department of Ophthalmology, Zealand University Hospital Roskilde, Roskilde, Denmark; 8 Division of Public Health, Department of Social Medicine, Graduate School of Medicine, Osaka University, Osaka, Japan; 9 Research Unit OPEN, Department of Clinical Research, University of Southern Denmark, Odense C, Denmark; 10 Department of Ophthalmology, Aalborg University Hospital, Aalborg, Denmark

**Keywords:** Bariatric surgery, Diabetes, Diabetic retinopathy, Epidemiology, Nationwide

## Abstract

**Aims:**

Bariatric surgery is used to induce weight loss and glycemic stability in type 2 diabetes (T2D). It has been a concern that this may lead to early worsening of diabetic retinopathy (DR) due to a rapid decline in HbA1c. In this study, we evaluated the risk of short and long-term DR development and need for ocular intervention in an entire nation of individuals with T2D undergoing bariatric surgery.

**Methods:**

The study comprised a national, register-based cohort of individuals with T2D screened for DR. Cases were matched by age, sex and DR level at the date of surgery (index date) with non-bariatric controls. We extracted information on DR levels, in- and outpatient treatments, pharmaceutical prescriptions and laboratory values. We evaluated worsening of DR (incident and progressive DR) at follow-up (6 and 36 months).

**Results:**

Amongst 238,967 individuals with T2D, who attended diabetic eye screening, we identified 553 that underwent bariatric surgery (0.2%) and 2677 non-bariatric controls. Median age was 49 years, and 63% were female. Cases had more comorbidities, lower HbA1c as well as more frequent use of glucose-lowering and antihypertensive medication than controls at index date. In a fully adjusted logistic regression model, the risk of DR worsening for cases was not significantly different compared to controls, neither short-term (OR 0.41 [CI 95% 0.13; 1.33], *p* = 0.14) nor long-term (OR 0.64 [CI 95% 0.33; 1.24], *p* = 0.18).

**Conclusions:**

In this nationwide study, bariatric surgery did not associate with increased risk of short- or long-term DR worsening.

**Supplementary Information:**

The online version contains supplementary material available at 10.1007/s00592-023-02140-w.

## Introduction

The global continuously increasing prevalence of type 2 diabetes has been described as a pandemic, surpassing 462 million affected individuals in 2017 and estimated to be the ninth leading cause of mortality [1]. Diabetic retinopathy (DR) is the most frequent complication to diabetes and a prominent cause of blindness [2]. Many risk factors have proved significant in relation to the development of DR, including hyperglycemia, hypertension, hyperlipidemia and obesity (all modifiable) as well as duration of diabetes and pregnancy (both non-modifiable) [3]. Bariatric surgery is a well-established medical intervention in patients with type 2 diabetes and severe overweight. In addition to significant weight loss it drastically changes the metabolic profile of the patient [4], including improvement in lipids and insulin sensitivity, and sometimes leading to diabetes remission post operatively [5]. The effect of bariatric surgery on retinal microvasculature is not well established, and existing research is not in agreement on the potential effects on DR. A systematic review and meta-analysis done in 2014 found a tendency towards progression of DR, probably due to a rapid decline in HbA1c levels post-surgery, which decreased as much as 3.9% (18.6 mmol/mol) compared to pre-surgery measurements [6]. However, more recent systematic reviews and meta-analyzes from 2017, found a better prognosis of DR, with lower incident rates and less progression compared to patients who did not undergo bariatric surgery [7, 8]. To our knowledge, no larger, register-based studies have examined the need for ocular intervention (laser treatment, intravitreal anti VEGF injection or vitrectomy) after bariatric surgery, but an observational study from 2016 found no instances where surgical intervention was warranted amongst their cohort [9].

Research on the subject is still inconclusive, with smaller study populations and lacking information regarding regression as well as the potential interventional consequences of DR progression, affecting patients with type 2 diabetes undergoing bariatric surgery. Hence, in this study, we aimed to explore the effect of bariatric surgery on DR development, in an entire population of individuals with type 2 diabetes, during a 3 year follow-up period.

## Methods

We performed a register-based matched cohort study utilizing the Danish registers. The cohort was identified in The Danish Registry of Diabetic Retinopathy (DiaBase), a national Danish clinical quality database, that holds information regarding all patients screened for DR in Denmark since 2013[10]. Data from various other national Danish registers were also included to enrich data; The Danish Civil Registration System [11] provided basic information on age, sex and civil status as well as enabled data linkage between registers due to the unique identification number (CPR number) given to all Danish inhabitants, The Danish National Patient Register [12] with diagnostic and treatment codes for in- and outpatient care, the Register of Laboratory Results for Research [13] with nationwide biochemical measurements and finally The Danish National Prescription Registry [14] that provided information on all prescribed and redeemed pharmaceuticals in Denmark.

The registers, utilized in this study, have been described in details by Grauslund et al. [15].

### Participants

As cases, we included patients registered in DiaBase with type 2 diabetes, above the age of 18 at index date, that had undergone any form of bariatric surgery from 2013 to 2022. Index date was set as the date of bariatric surgery defined by the registration of a KJDF* (gastric bypass, gastric banding and gastric sleeve) ICD-10 surgical code. Patients registered with a KJDF* code prior to 2013, were excluded from the case population. The control group was selected amongst the remaining DiaBase population with type 2 diabetes, with no history of bariatric surgery, matched to cases by sex, age (year of birth) and level of DR at index date (Table 1). For screening specific outcomes, patients with fewer than 2 screening episodes, were excluded from both case and control groups.

**Table 1:** Baseline characteristics for patients with type 2 diabetes with (cases) and without (controls) bariatric surgery at index date

	All	Bariatric surgery		*P* value
	n = 3230	Yes (cases) n = 553	No (controls) n = 2677	
Sex, % female	2032 (62.9%)	348 (62.9%)	1684 (62.9%)	0.99
Age, years (IQR)	49 (42–55)	49 (42–55)	49 (42–55)	0.50
Duration of diabetes, years (IQR)	6.09 (2.4–11.1)	5.10 (1.9–9.9)	6.22 (2.57–11.35)	< 0.001
Marital status, n (%)				0.55
Never married	981 (30.4%)	163 (29.5%)	818 (30.6%)	
Married	1732 (53.6%)	293 (52.9%)	1439 (53.8%)	
Widowed or divorced	517 (16.0%)	97 (17.5%)	420 (15.7%)	
CCI score, n (%)				0.002
0 (low)	2536 (78.5%)	406 (73.4%)	2130 (79.6%)	
1 (moderate low)	432 (13.4%)	90 (16.3%)	342 (12.8%)	
2 (moderate high)	195 (6.0%)	48 (8.7%)	147 (5.5%)	
≥ 3 (high)	67 (2.1%)	9 (1.6%)	58 (2.2%)	
Level of DR, n (%)*				0.40
0 (no DR)	2878 (89.1%)	487 (88.1%)	2391 (89.3%)	
1 (mild NPDR)	209 (6.5%)	36 (6.5%)	173 (6.5%)	
2 (moderate NPDR)	101 (3.1%)	18 (3.3%)	83 (3.1%)	
3 (severe NPDR)	22 (0.7%)	6 (1.1%)	16 (0.6%)	
4 (PDR)	20 (0.6%)	6 (1.1%)	14 (0.5%)	
BMI, n (%)				
Class I obesity (BMI 30–34.9)	12 (2.2%)	12 (2.2%)	NA	
Class II obesity (BMI 35–39.9)	119 (21.6%)	119 (21.6%)	NA	
Class III obesity (BMI 40–55 +)	195 (35.3%)	195 (35.3%)	NA	
Undefined overweight	226 (40.9%)	226 (40.9%)	NA	
HbA1c, median [IQR]*	6.9% (52 [45–62])	6.5% (48 [42–55])	7.0% (53 [46–63])	< 0.001
Pharmacological treatment*				
Glucose-lowering medication				
GLP-1	838 (25.9%)	274 (49.6%)	564 (21.1%)	< 0.001
SGLT-2	483 (14.9%)	98 (17.7%)	385 (14.4%)	0.045
Metformin	2337 (72.4%)	454 (82.1%)	1883 (70.3%)	< 0.001
Insulin	939 (29.1%)	156 (28.2%)	783 (29.3%)	0.62
Antihypertensive medication n (%)	1920 (59.4%)	406 (73.4%)	1514 (56.6%)	< 0.001
Cholesterol lowering medication n (%)	2019 (62.5%)	354 (64.0%)	1665 (62.2%)	0.42

Results are given in counts (n) or medians with percentages (%) or interquartile range (IQR). BMI was only available for cases, as it is not measured routinely for patients not undergoing bariatric surgery

*Index date* date of bariatric surgery, *CCI* Charlson comorbidity index, *DR* diabetic retinopathy, *NPDR* non proliferative DR, *PDR* proliferative DR, *BMI* body mass index, *HbA1c* glycated hemoglobin, *GLP-1* glucagon Like Peptide 1, *SGLT-2* selective sodium glucose co transporter 2

^*^Closest measurement/registration prior to index date (within 1 year)

### Outcomes

Our main outcome was DR worsening (quantifiable at screening visits) in which incident (DR level 1–4 at follow-up) and progressive DR (≥ two-step progression or progression to PDR) were pooled and assessed at month 6 and month 36, as well as the need for post-surgical ocular intervention (panretinal or focal photocoagulation, intravitreal injections or vitrectomy) assed within 1 year and after 1 year of surgery. DR improvement (≥ two-step regression) during follow-up was also evaluated at month 6 and 36. Finally, we examined changes in pharmaceutical treatments as well as biochemical measurements amongst cases and controls during follow-up.

### Covariates

From DiaBase, we extracted information on screening dates and level of DR at each screening (ICDR scale [16], 0–4 [0 = no DR, 1 = mild non proliferative DR (NPDR), 2 = moderate NPDR, 3 = severe NPDR and 4 = proliferative DR (PDR)]).

From The Danish Civil Registration System we used age, sex (female or male), and marital status (married/cohabitating, single or divorced).

The Danish National Patient Register provided information on surgical interventions (bariatric surgery [KJDF*], vitrectomy [(KCKD65 and DH334B) or (KCKD65 and DH431 and DH36*)], intravitreal anti VEGF injection [(KCKD05B) and not (DH34* or DH353*) within 6 months prior to injection], panretinal [KCKC15 and not DH34* within 6 months prior to injection] and focal photocoagulation [KCKC10 and DH36*]) as well as systemic illnesses used to calculate a modified (excluding diabetes) Charlson Comorbidity Index score (CCI score) 5 years prior to index date (0 [low], 1 [moderate low], 2 [moderate high] and ≥ 3 [high]). The register also provided the diagnostic codes for the classification of bodyweight (unspecified overweight [DE660, DE660A, DE668 and DE669], obesity grade 1 [DE660B], obesity grade 2 [DE660C] and obesity grade 3 [DE660E, D660F, D660G and D660H]), from which a marker of BMI was constructed.

From the Register of Laboratory Results for Research we extracted information on laboratory values for measurements of hemoglobin A1c (HbA1c [% (mmol/mol)]), plasma creatinine (P-crea [µmol/L]), albumin/creatinine ratio in urine (uACR [mg/g]), estimated glomerular filtration rate (eGFR [mL/minute/1.73 m^2^]), low density lipoprotein cholesterol (LDL [mmol/L]), total cholesterol (mmol/L), high density lipoprotein cholesterol (HDL [mmol/L] and triacylglyceroles (TG [mmol/L]).

Finally, we utilized The Danish National Prescription Registry, from which information on prescribed and redeemed medications (antihypertensive-, antidiabetic- [GLP1 analogues, SGLT2 inhibitors, insulins and non-insulins] and lipid lowering medications) was used.

To differentiate between patients according to type of diabetes (type 1 and type 2 diabetes), we examined patients’ diagnosis- and pharmaceutical codes from The Danish National Patient Register and The Danish National Prescription Registry and divided them using an endocrinologist recommended algorithm “Appendix”.

### Statistical methods

All data analyzes were performed with Stata 17.0 (StataCorp LLC., College Station, Texas, USA). Data are presented descriptively with medians and interquartile ranges (IQR) or counts and percentages. Statistical significance was defined as *p*-values < 0.05 and confidence intervals not including 1. In Table 1, Wilcoxon rank-sum and Pearson's chi-square test were used for continuous and categorical variables, respectively, to determine possible differences between cases and controls. To examine the relation of bariatric surgery and DR (worsening and improvement), semi-adjusted (age and sex) and fully adjusted (age, sex and all significant differences in Table 1) multiple logistic regression models resulting in odds ratios (OR) were used (Table 3). A Cox regression model resulting in hazard ratios (HR) with same adjustment steps was used for examining post-surgical ocular intervention. OR were calculated at fixed post-surgery timepoints to assess a potential transient worsening (month six ± 3 months), and also a more long-term effect (month 36 ± 9 months) using the screening date closest to these. HR for ocular intervention were calculated short-term (index date till month 12) and long-term (month 12 till end of follow-up). To utilize data from both eyes, clustered standard errors were applied to all regression models. Cases were matched to controls with replacements and we aimed for a case control ratio of 1:5, but as some cases were matched to fewer controls due to the demands of the matching criteria, a final ratio of 1:4.8 was obtained. In cases where missing data were present, and exceeded acceptable levels, multiple imputation (MI) was used when appropriate, determined by the type of missing data (missing completely at random, missing at random or missing not at random), which was evaluated both statistically and logically using preexisting, established knowledge of covariates and how they were obtained in clinical settings.

## Results

Among 238,967 patients with type 2 diabetes attending the Danish screening program for DR from 2013 to 2022, we identified 553 cases who underwent bariatric surgery during follow-up and matched them to 2677 non-bariatric controls. Included individuals were primarily female (62.9%) and had a median age of 49 years (IQR 42–55 years), they had a higher CCI score (moderate low [16.3 vs. 12.8%] and moderate high [8.9 vs. 5.5%], *p* < 0.01), shorter duration of diabetes (5.1 vs. 6.2 years, *p* < 0.01), better glycemic stability (HbA1c 6.5% vs. 7.0% [48.0 vs. 53.0 mmol/l], *p* < 0.01) as well as more frequent use of metformin (82.1 vs. 70.3%, < 0.01), antihypertensive medications (73.4 vs. 56.6%, < 0.01), GLP-1 analogues (49.5 vs. 21.1%, *p* < 0.01) and SGLT-2 inhibitors (17.7 vs. 14.4%, *p* = 0.04) than controls at index date. They did not differ in regards to marital status, use of insulin or cholesterol lowering medications (Table 1).

DR worsening (incident DR and progressive DR pooled) at 6 and 36 months was seen in 2.9% and 5.2% of cases and 8.4% and 7.9% of controls (Table 2). Odds for short and long-term DR worsening after bariatric surgery were OR 0.32 (CI 95% 0.12–0.84, *p* = 0.02) and OR 0.68 (CI 95% 0.35–1.33, *p* = 0.26) in the semi adjusted model (Table 3). In the fully adjusted model with MI for missing HbA1c values (9.4% and 13.7% of cases and controls respectively), results for short- and long-term worsening were OR 0.41 (CI 95% 0.13–1.33, *p* = 0.14) and OR 0.71 (CI 95% 0.34–1.46, *p* = 0.35) respectively (Table 3). We found no accounts of either short- or long-term ocular treatment needs post-surgery in our case population except for < 5 cases of intravitreal injections (too few events to statistically analyze). We performed a post-hoc analysis stratified by pre-existing DR at index date to examine whether this impacted the odds of DR development, however we found no increased odds of DR worsening at any point, in either group (Supplementary table 2).

**Table 2:** Post-surgical DR worsening and improvement events in cases (with bariatric surgery) and controls (without bariatric surgery) at 6 and 36 months

	Cases eligible for analysis (%)	Controls eligible for analysis (%)	6 months				36 months			
			Eligible cases with screening at 6 months (± 3 months)	Events, cases (%)	Eligible controls with screening at 6 months (± 3 months)	Events, controls (%)	Eligible cases with screening at 36 months (± 9 months)	Events, cases (%)	Eligible controls with screening at 36 months (± 9 months	Events, controls (%)
DR improvement	44 (22.1%)	155 (77.9%)	11 (25.0%)	< 5	76 (49.03%)	15 (19.7%)	9 (20.5%)	6 (66.7%)	68 (43.9%)	25 (36.8%)
DR worsening	1095 (17.1%)	5315 (82.9%)	274 (25.0%)	8 (2.9%)	404 (7.6%)	34 (8.4%)	249 (22.7%)	13 (5.2%)	2131 (40.1%)	169 (7.9%)

DR improvement was defined as 2 step improvement of DR. DR worsening was defined as incident, 2-step-progression or progression to proliferative DR (PDR). Criteria of eligibility for analysis of improvement was DR level 2 or 3 at baseline. Criteria for eligibility for analysis of DR worsening was DR level 0 at baseline and DR level 1–4 at follow-up (incident DR), DR level 0–2 at baseline and DR level 2–4 at follow-up (DR progression) or DR level 0–3 at baseline and DR level 4 at follow-up (progression to PDR)

*DR* diabetic retinopathy

**Table 3:** Multiple regression analysis with short- and long-term odds of DR improvement and DR worsening in cases (with bariatric surgery) compared to controls (without bariatric surgery)

	Short-term				Long-term			
	Semi adjusted		Fully adjusted		Semi adjusted		Fully adjusted	
	Adjusted OR	*P* value	Adjusted OR	*P* value	Adjusted OR	*P* value	Adjusted OR	*P* value
DR improvement	1.53 (0.23; 10.19)	0.66	1.25 (0.07; 21.67)	0.88	3.44 (0.48; 24.41)	0.22	3.25 (0.31; 34.00)	0.33
DR worsening	0.33 (0.12; 0.86)	0.02	0.41 (0.13; 1.33)	0.14	0.64 (0.33; 1.24)	0.18	0.71 (0.34; 1.46)	0.35

Short-term = 6 months ± 3 months. Long-term = 36 months ± 9 months. Semi adjusted = sex and age adjusted. Fully adjusted = adjusted for age, sex and all significant factors in Table 1 with multiple imputations (MI) for HbA1c. DR improvement was defined as 2 step improvement of DR. DR worsening was defined as incident, 2-step-progression or progression to proliferative DR (PDR)

*HbA1c* glycated hemoglobin, *DR* diabetic retinopathy

**Fig. 1:**
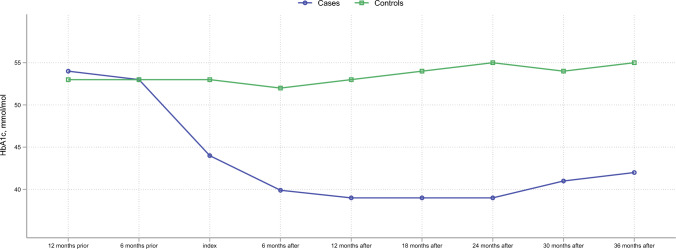
Median HbA1c measurements in mmol/mol at fixed time points from 12 months prior to bariatric surgery until 36 months post-surgery for cases (with bariatric surgery) and at equal points for controls (without bariatric surgery)

We examined biochemical measurements (HbA1c, lipids and nephrology) as well as medication use (insulin, non-insulin glucose lowering, antihypertensive and lipid lowering medication) pre- and post-surgery (short- and long-term) to evaluate how bariatric surgery affected these parameters (Supplementary Table 1). In pre-surgical measurements, glycemic stability was best amongst cases and a significant drop in HbA1c was seen leading up to and directly following surgery (Fig. 1). Although a slight increase in HbA1c was seen in cases long-term compared to directly after surgery, the levels still remained lower than pre-surgical measurements, and were lower than control individuals levels at all times (6.5 vs. 7.0% [48.0 vs. 53.0 mmol/mol] *p* < 0.001, 5.8 vs. 6.9% [39.8 vs. 52.0 mmol/mol] *p* < 0.001 and 5.9 vs. 7.2% [41.0 vs. 55.0] *p* < 0.001) (Supplementary table 1). Plasma TG levels were reduced in cases after surgery, and stayed below the upper recommended limit for patients with diabetes, whereas TG levels in controls stayed high (1.98 vs. 1.80 mmol/l; *p* < 0.001, 1.29 vs. 1.81 mmol/l; *p* < 0.001 and 1.40 vs. 1.76 mmol/l; *p* < 0.001) throughout follow-up. Kidney function measured by glomerular filtration (eGFR), plasma creatinine and urine albumin/creatinine ratio (uACR) were within normal limits during the follow-up time, in both groups. Due to a detected interaction between bariatric surgery and BMI in regards to DR development, we also performed a stratified analysis according to BMI at 36 months post-surgery. This showed no difference in odds of DR worsening or ocular intervention, no matter the degree of overweight.

## Discussion

In this nationwide study examining the risk of DR worsening amongst individuals with type 2 diabetes who underwent bariatric surgery, we did not find evidence indicating signs of post-surgical transient or long-term worsening of DR, nor an increased need for ophthalmological intervention. Regression analysis suggested lower or equal rates of DR worsening in individuals who underwent bariatric surgery compared to persons who did not.

This positive outcome aligns with several recent, smaller studies suggesting that bariatric surgery has no detrimental effect on the development of DR in patients with type 2 diabetes [7, 8]. Our nationwide data showed good baseline glycemic stability amongst our case population, as well as a pre-surgical decline in HbA1c followed by a further post-surgical decrease in HbA1c, which might explain the low rates of progression. Previous studies disagree on the role of pre-surgical HbA1c levels in regards to DR progression; with one study showing that a higher baseline HbA1c with a significant post-surgical drop increased the risk of DR progression [17], another study did not find an association [18]. The rates of DR worsening were also lower than seen amongst the general screening population of patients with type 2 diabetes in the Danish screening program [19] suggesting that patients eligible for bariatric surgery are following the pre-surgical guidelines promoting good pre-surgical glycemic stability, weightloss and lifestyle changes, amongst other initiatives, all intended to ensure optimal results of surgery [20]. Another reason for good glycemic stability, and generally acceptable biochemical measurements amongst our population as a whole, might be the effectiveness of the screening program itself alongside other healthcare appointments at the patients primary care physician; discovering tendencies towards worsening in DR or irregularities in systemic examinations and bloodwork, thus being able to induct initiatives to improve glycemic stability, and in turn halt further progression. Finally, our case population had a shorter diabetes duration than our control population which might also be in their favor, considering the known association between diabetes duration and DR development [21]. The low progression rates support the virtually non-existent need for ocular intervention post-surgery, where no differences were detected between cases and controls, as ocular intervention is tied to PDR and progression was seen in 2.9% and 8.4% of cases and controls, respectively. Nutritional deficits following malabsorptive bariatric surgery is well known and vitamin D deficiency has been associated with DR worsening [22]. Lower levels of vitamin A could cause several ophthalmic issues such as nyctalopia, corneal and conjunctival xerosis leading to ulcerations might also be of concern [23], especially in patients with diabetes, with decreased corneal sensitivity. We were not able to conduct any sub-group analysis where cases were stratified by type of bariatric surgery, due to insufficient data on specific type’s bariatric procedures. When stratified by DR level at index date no increased odds were seen in either group, which is in accordance with previous findings [8], however the overall small number of events in our study must be taken in to consideration. We found a significant postoperative drop in plasma TG amongst our case population, which is in accordance with previous studies [24–26].

Our study was strengthened by the vast amount of register-based data, linked throughout several registers, providing a representative sample of the majority of the Danish population with type 2 diabetes and with an established high level of completeness [14, 27]. The long-term follow-up time was also a strength, as post-surgical changes in DR potentially could be more long-term, due to the effect of post-prandial hypoglycemia seen in some patients after bariatric surgery [28].

We must also address some limitations to the study, starting with BMI measurements. BMI (or height and weight) is not routinely registered in any nationwide Danish registers, so a marker using ICD-10 codes had to be constructed, and measurements were only available for the case population. We did not have access to information on lifestyle factors such as smoking status, alcohol consumption and other dietary choices. Another limitation to consider is that our study relied on a screening database, which means that we did not have access to data from individuals with diabetes who never attended the DR screening program and the available data for screening specific outcomes are limited to the dates of screening.

In conclusion, this population-based study adds support to the claim that bariatric surgery is safe in regards to patients with type 2 diabetes. In our population of patients, with overall good glycemic stability, undergoing bariatric surgery, we did not observe increased odds of short- or long-term DR worsening or need for ophthalmic intervention, regardless of pre-existing DR at index date.

### Electronic supplementary material

Below is the link to the electronic supplementary material.

Supplementary file1 (DOCX 19 kb)

## Data Availability

The datasets analyzed in the current study are available from the Danish Health Data Authority, but restrictions apply to the availability of these data, which were used under license from OPEN and the Danish Health Data Authority and thus are not publicly available.

## Appendix

Diabetes classification in the Danish registers developed by the Ocular and Systemic complications In diabetic retinopathy (OASIS) study group

In a generic, non-selected population (National Patient Registry).

**Table:**

Type 1 diabetes	Latest given diagnostic code must be DE10
	AND
	First prescription of A10A within a year of first DE10 diagnosis*
	AND
	Last prescription of A10A within a year of exit
	AND number of prescriptions ≥ number of years from first prescription to exit
Type 2 diabetes	Diagnostic code DE11
	AND
	≥ two A10B prescriptions**
	OR
	≥ two DE11 diagnostic codes
	OR
	≥ two prescriptions of A10A if age 40 + at prescription
	OR
	≥ two prescriptions of A10B if age 30 + at prescription
	*Exclusions:*
	*Already grouped as type 1 diabetes*
	*OR/AND*
	*Female AND diagnostic code for PCOS (E282) AND no diagnostic code for diabetes type II (DE11)*

In a population consisting exclusively of patients presumed to have diabetes (DiaBase).

**Table:**

Type 1 diabetes	Latest given diagnostic code in The National Patient Register = DE10*
	AND
	First prescription of A10A within a year of first DE10 diagnosis
	AND
	Last prescription of A10A within a year of exit
	AND number of prescriptions ≥ number of years from first prescription to exit
Type 2 diabetes	The remaining population

*DiaBase* Danish registry of diabetic retinopathy

*Since data from The Danish National Prescription Registry is available from 1995 and onward, patients with a diagnosis given before this year and prescriptions starting in 1995, could be excluded unnecessarily. In the case of diagnosis given before 1995, the first prescription must therefore be in 1995

**One prescription is allowed for patients who received their diagnosis during 2022

## Abbreviations

BMI: Body mass index
CCI: Charlson comorbidity index
DR: Diabetic retinopathy
DiaBase: The Danish registry of diabetic retinopathy
GLP1: Glugacon like peptide 1
ICDR: International clinical diabetic retinopathy severity scale
MI: Multiple imputation
NPDR: Non proliferative DR
OPEN: Open patient data explorative network
PDR: Proliferative DR
SGLT2: Selective sodium glucose co transporter
TG: Triacylglycerol
uACR: Urine albumin/creatinine ratio
