# Supplementary material for: Bariatric surgery in individuals with type 2 diabetes is not associated with short or long-term risk of diabetic retinopathy progression: results from a nationwide cohort study
Source: Acta Diabetol. 2023 Jul 8;60(11):1531–9. doi: 10.1007/s00592-023-02140-w (PMC10520211; doi:10.1007/s00592-023-02140-w)
Supplement: Supplementary file 1 — Supplementary file1 (DOCX 19 kb) [file 592_2023_2140_MOESM1_ESM.docx]

Supplementary Table 1 – Biochemical measurements and pharmaceutical treatments for cases (with bariatric surgery) and controls (without bariatric surgery) at equal time-points

Results are given in counts (n) or medians with percentages (%) or interquartile range (IQR). HbA1c given in NGSP (%) and mmol/mol. Pre-surgery = closest registration prior to surgery date (within one year). Short-term = closest measurement/registration to 6 months. Long-term = closest measurement/registration to 36 months. HbA1c = glycated hemoglobin, HDL = high density lipoprotein, LDL = low density lipoprotein, eGFR = glomerular filtration rate, uACR = urine albumin/creatine ratio, IQR = interquartile range.

|  | **Pre-surgery** | | | | |  | **Short-term** | | | | |  | **Long-term** | | | | |
| --- | --- | --- | --- | --- | --- | --- | --- | --- | --- | --- | --- | --- | --- | --- | --- | --- | --- |
|  | **Cases** | | **Controls** | |  |  | **Cases** | | **Controls** | |  |  | **Cases** | | **Controls** | |  |
|  | Individuals with measurement/medication | Median (IQR) | Individuals with measurement/medication | Median (IQR) | P value |  | Individuals with measurement/medication | IQR cases | Individuals with measurement/medication | Median (IQR) | P value |  | Individuals with measurement/medication | Median (IQR) | Individuals with measurement/medication | Median (IQR) | P value |
| **BIOCHEMICAL** |  |  |  |  |  |  |  |  |  |  |  |  |  |  |  |  |  |
| HbA1c | 501 (0.91) | 6.5 [6.0;7.2]  (48.00 [42.00;55.00]) | 2324 (4.20) | 7.0 [6.4;7.9] (53.00 [46.00;63.00]) | <0.001 |  | 467 (0.84) | 5.8 [5.4;6.4] (39.80 [36.00;46.00]) | 1939 (3.51) | 6.9 [6.4;7.9]  (52.00 [46.00;63.00]) | <0.001 |  | 335 (0.61) | 5.9 [5.4;6.5]  (41.00 [36.00;48.00]) | 1794 (3.24) | 7.2 [6.5;8.1]  (55.00 [47.70;65.00]) | <0.001 |
| **Lipids** |  |  |  |  |  |  |  |  |  |  |  |  |  |  |  |  |  |
| Triacylglyceroles | 484 (0.88) | 1.98 (1.47;2.70) | 2181 (3.94) | 1.80 (1.20;2.66) | <0.001 |  | 400 (0.72) | 1.29 (0.97;1.70) | 1427 (2.58) | 1.81 (1.22;2.74) | <0.001 |  | 307 (0.56) | 1.40 (1.04;2.00) | 1701 (3.08) | 1.76 (1.20;2.60) | <0.001 |
| HDL | 488 (0.88) | 1.00 (0.90;1.20) | 2184 (3.95) | 1.10 (0.95;1.40) | <0.001 |  | 402 (0.73) | 1.20 (1.00;1.40) | 1430 (2.59) | 1.10 (0.95;1.40) | 0.23 |  | 306 (0.55) | 1.30 (1.10;1.60) | 1701 (3.08) | 1.20 (1.00;1.40) | <0.001 |
| LDL | 477 (0.86) | 2.10 (1.60;2.70) | 2112 (3.82) | 2.10 (1.60;2.70) | 0.88 |  | 401 (0.73) | 2.10 (1.50;2.60) | 1371 (2.48) | 2.00 (1.60;2.70) | 0.21 |  | 302 (0.55) | 2.20 (1.70;2.70) | 1651 (2.99) | 2.00 (1.50;2.60) | <0.001 |
| Total Cholesterol | 489 (0.88) | 4.00 (3.40;4.80) | 2191 (3.96) | 4.20 (3.60;4.90) | 0.01 |  | 401 (0.73) | 3.90 (3.20;4.40) | 1439 (2.60) | 4.10 (3.60;4.90) | <0.001 |  | 308 (0.56) | 4.30 (3.70;4.80) | 1708 (3.09) | 4.10 (3.50;4.80) | 0.07 |
| **Nephrology** |  |  |  |  |  |  |  |  |  |  |  |  |  |  |  |  |  |
| eGFR | 502 (0.91) | 90.00 (82.00;90.00) | 2249 (4.07) | 90.00 (88.00;90.00) | <0.001 |  | 433 (0.78) | 90.00 (89.00;90.00) | 1707 (3.09) | 90.00 (88.00;90.00) | 0.78 |  | 337 (0.61) | 90.00 (89.00;90.00) | 1776 (3.21) | 90.00 (89.00;90.00) | 0.76 |
| Plasma creatinine | 514 (0.93) | 68.00 (59.00;79.00) | 2318 (4.19) | 64.00 (55.00;75.00) | <0.001 |  | 454 (0.82) | 64.00 (57.00;74.00) | 1783 (3.22) | 65.00 (55.00;76.00) | 0.85 |  | 346 (0.63) | 64.00 (55.00;74.00) | 1792 (3.24) | 64.00 (55.00;76.00) | 0.99 |
| uACR | 293 (0.53) | 13.00 (7.00;34.00) | 1503 (2.72) | 10.00 (5.00;28.00) | <0.001 |  | 181 (0.33) | 9.00 (5.00;19.00) | 835 (1.51) | 11.00 (6.00;32.00) | 0.01 |  | 178 (0.32) | 11.00 (6.10;27.00) | 1289 (2.33) | 11.00 (6.00;29.00) | 0.89 |
| **MEDICATION** |  |  |  |  |  |  |  |  |  |  |  |  |  |  |  |  |  |
| Insulin | 156 (0.28) |  | 783 (1.42) |  | 0.62 |  | 51 (0.09) |  | 727 (1.31) |  | <0.001 |  | 37 (0.07) |  | 659 (1.19) |  | <0.001 |
| Non-insulin glucose lowering medication | 501 (0.91) |  | 2009 (3.63) |  | <0.001 |  | 186 (0.34) |  | 1878 (3.40) |  | <0.001 |  | 155 (0.28) |  | 1498 (2.71) |  | <0.001 |
| Antihypertensive medication | 406 (0.73) |  | 1514 (2.74) |  | <0.001 |  | 283 (0.51) |  | 1421 (2.57) |  | 0.41 |  | 211 (0.38) |  | 1191 (2.15) |  | 0.01 |
| Lipid lowering medication | 354 (0.64) |  | 1665 (3.01) |  | 0.42 |  | 230 (0.42) |  | 1530 (2.77) |  | <0.001 |  | 171 (0.31) |  | 1357 (2.45) |  | <0.001 |

| DR 0 | Cases, n (%) | Controls, n (%) | Event cases, n(%) | Event controls, n(%) | Adjusted OR | P-value |
| --- | --- | --- | --- | --- | --- | --- |
| 6 months | 123 (25.26%) | 130 (5.44%) | 5 (4.07%) | 17 (13.08%) | 0.26 (0.09; 0.74) | 0.01 |
| 36 months | 110 (22.59%) | 933 (39.02%) | 9 (8.18%) | 108 (11.58%) | 0.74 (0.36; 1.51) | 0.41 |
|  |  |  |  |  |  |  |
| DR 1-4 | **Cases, n (%)** | **Controls, n (%)** | **Event cases, n(%)** | **Event controls, n(%)** | **Adjusted OR** | **P-value** |
| 6 months | 14 (22.58%) | 74 (27.01%) | <5 | 6 (8.11%) | 0.81 (0.08; 8.35) | 0.86 |
| 36 months | 16 (25.81%) | 138 (50.36%) | <5 | 17 (12.32%) | 0.95 (0.20; 4.51) | 0.95 |

Supplementary table 2 – Multiple regression analysis stratified by preexisting DR (DR vs. no DR) at index date for cases (with bariatric surgery) and controls (without bariatric surgery) short- and long-term.

Short-term = 6 months ± three months. Long-term = 36 months ± nine months. Adjusted = sex and age adjusted. DR = diabetic retinopathy. DR worsening was defined as incident, 2-step-progression or progression to proliferative DR (PDR).
